# Long-Term Complications after Surgical or Medical Treatment of Predominantly Forefoot Diabetic Foot Osteomyelitis: 1 Year Follow Up

**DOI:** 10.3390/jcm10091943

**Published:** 2021-05-01

**Authors:** Aroa Tardáguila-García, Yolanda García-Álvarez, Esther García-Morales, Mateo López-Moral, Irene Sanz-Corbalán, José Luis Lázaro-Martínez

**Affiliations:** Diabetic Foot Unit, Clínica Universitaria de Podología, Facultad de Enfermería, Fisioterapia y Podología, Instituto de Investigación Sanitaria del Hospital Clínico San Carlos (IdISSC), Universidad Complutense de Madrid, 28040 Madrid, Spain; aroa_tg@hotmail.com (A.T.-G.); esthergarciamorales@yahoo.es (E.G.-M.); matlopezmor@hotmail.com (M.L.-M.); irsanz01@ucm.es (I.S.-C.); diabetes@ucm.es (J.L.L.-M.)

**Keywords:** diabetic foot osteomyelitis (DFO), complications, medical treatment, surgical treatment

## Abstract

Aim: To compare long-term complications according to the treatment received for management of diabetic foot osteomyelitis (surgical or medical) at 1 year follow up. Design and Participants: A prospective observational study was conducted involving 116 patients with diabetic foot osteomyelitis. The patients received surgical or medical treatment based on the principles described in the literature. To register the development of a complication, both groups of treatments were followed-up 1 year after the ulcer had healed. Results: Ninety-six (82.8%) patients received surgical treatment and 20 (17.2%) medical treatment. No differences were found in the time to healing between both groups of treatment, 15.7 ± 9.2 weeks in the surgical group versus 16.4 ± 12.1 weeks in the medical group; *p* = 0.103. During follow up, 85 (73.3%) patients developed complications without differences between both groups, 68 (70.8%) in the surgical group versus 17 (85%) in the medical group (*p* = 0.193). The most common complication in both groups was re-ulceration. We did not observe significant differences comparing complication-free time survival between both treatments (*p* = 0.665). Conclusion: The onset of complications after healing in patients who suffered from diabetic foot osteomyelitis was not associated with the treatment received. Surgical and medical approaches to the management of diabetic foot osteomyelitis produced similar results in long-term follow up.

## 1. Introduction

Diabetic foot osteomyelitis (DFO) is a severe complication of diabetic foot disease [1]. DFO affects 50–60% of severe diabetic foot infections and approximately 20% of moderate infections [2].

The best treatments for DFO, surgery or antibiotics, are still a matter of debate [3,4]. Recommendations included in international guidelines [5,6] for the management of patients with DFO can vary widely, due to the various professionals who treat DFO patients, who have differing points of view in the selection of antibiotics or surgical techniques [7]. On the one hand, we find clinicians who defend the use of medical treatment in DFO management [8,9,10]. On the other hand, some clinicians defend the use of surgery to treat DFO [4,11]. Nevertheless, there is a certain consensus in the primary selection of treatment in patients with DFO based on the characteristics of the patient and the ulcer [12].

Most publications addressing the management of DFO have mainly investigated healing rates, time to healing, and short-term complications (especially major or minor amputation) [3,12,13,14,15,16,17]. Short-term complications and disadvantages associated with the treatment of DFO have been well described, for instance, transfer lesion in patients who received surgical treatment and bacterial resistance in patients who received medical treatment [12]. There have been few studies with long-term follow up [18], and there are no studies that have compared both treatments prospectively, so there is a lack of investigations that explore what happens to DFO patients during long-term follow up.

The aim of the study was to compare long-term complications at 1 year follow up according to the treatment received for the management of DFO (surgical or medical).

## 2. Materials and Methods

A prospective observational study was conducted between November 2014 and November 2018, including 116 patients undergoing DFO management either by surgical or medical treatment at a specialised diabetic foot unit.

We included patients with diabetes type 1 and 2, aged >18 years, having ulcers with an area between 1 and 5 cm^2^, who received surgical or antibiotic treatment for the management of DFO and demonstrated healing of the ulcer and who agreed to be included in the study after providing written consent. Patients were excluded in case of extensive or necrotising soft tissue infection, critical limb ischemia (CLI), acute Charcot foot, women who were pregnant or lactating and those who refused to be included in the study.

During the baseline visit, we collected the medical history and demographic characteristics of the patient; the ulcer area was evaluated by planimetry using a wound measurement system (VISITRAK™; Smith & Nephew, Hull, UK), confirmed DFO diagnosis and assessed by vascular and neurological examination.

A DFO diagnosis was established by a combination of PTB test and X-ray (two standard views) [19]. The PTB test was performed using Halsted mosquito forceps and was considered positive when the researcher could feel a hard or gritty surface. An X-ray was considered positive for osteomyelitis if it showed cortical disruption, periosteal elevation, a sequestrum or involucrum, or gross bone destruction. Diagnostic confirmation was obtained by bone culture or histology [6,20].

During the vascular examination, peripheral arterial disease (PAD) was diagnosed if the patient met the following criteria: the absence of both distal pulses (dorsalis pedis and posterior tibial) and ankle–brachial index (ABI) < 0.9. In patients with ABI > 1.4, we considered PAD with a toe–brachial index < 0.7 and a transcutaneous oxygen pressure < 30 mmHg, using a TCM4 transcutaneous monitor (Radiometer Medical, Brønshøj, Denmark) [21,22]. CLI was diagnosed if the patient met the following criteria: the absence of both distal pulses and ankle pressure < 70 mmHg, ABI < 0.5 or a toe systolic pressure < 50 mmHg [22,23].

Neurological examinations were conducted by a Semmes–Weinstein 5.07/10 g monofilament (Novalab Ibérica, Alcal. de Henares, Madrid, Spain) and a biotensiometer (Me.Te.Da. S.r.l., San Benedetto del Tronto, Italy). Neuropathy was diagnosed in patients who felt nothing during one of the two tests [24].

The patients received either surgical or medical treatment, according to the previously published recommendations [15]: Surgical treatment was indicated for patients with foot infection associated with substantial bone necrosis, patients with a particularly high risk for antibiotic-related problems, DFO with infecting pathogens that were resistant to available antibiotics, lower limbs suffering from untreatable ischemia that precluded systemic antibiotic delivery and when the patient had a strong preference for surgery [15]. The surgery group underwent conservative surgery, defined as procedures in which only infected bone and non-viable soft tissue are removed, but no amputation of any part of the foot is undertaken [4]. All surgeries were performed by the same surgeon (JLM), and bone samples were sent for microbiology and/or pathology analysis for diagnostic confirmation of DFO. During the first week after surgery, all patients received antibiotic treatment according to the results of the antibiogram [25]; in cases of negative culture results, we prescribed empirical antibiotics according to IDSA guidelines [5].

Medical treatment was indicated for patients too medically unstable for surgery: patients with a likely poor postsurgical foot mechanics, patients with an infection limited to small forefoot lesion, when the cost of surgery was prohibitive for the patient and when the patient had a strong preference to avoid surgical intervention. The medical group underwent medical treatment consisting of empiric antibiotic treatment at the beginning [5] and later modified according to the results of the bone culture [25]. Antibiotic treatment lasted 90 days [3,26]. Patients included in the medical group did not receive debridement of the affected bone.

Patients were evaluated twice per week until healing. According to the wound care protocol of our department, both groups received the same local treatment, selecting the dressing based on exudate control, comfort and cost. A removable cast walker was used as off-loading for every patient.

Once the ulcer healed, defined as complete epithelialisation without any drainage of a previous foot ulcer site after 2 weeks of the ulcer closure [27] and according to the international recommendations [28], the patients wore customised insoles and therapeutic footwear, and they were monitored monthly by a podiatrist. Patients were asked once per month about their compliance. After healing, we performed the follow-up visits over 1 year: visit 1 (ulcer healed); visit 2 (1 month after ulcer healed); visit 3 (6 months after ulcer healed); and visit 4 (12 months after ulcer healed). During follow-up visits, we registered the complications that the patients suffered. All subjects were followed until the end of the follow-up time, except in cases of any adverse event that caused premature termination in the study or death. We recorded the following outcomes as a complication event: DFO recurrence, bone reinfection; new case of DFO, new bone infection; soft tissue infection; infection of the skin, which can spread to deeper structures, without affecting the bone; ulcer recurrence, new ulcer located in the same location where the ulcer under study appeared; re-ulceration, new ulcer irrespective of the location where the ulcer under study appeared; minor amputation, any resection through or distal to the ankle; major amputation, any resection proximal to the ankle; death; other complications and events related to diabetic foot syndrome.

Ethical approval was obtained, and the study was completed in accordance with the ethical standards of the responsible committee (14/485-E). Informed consent was obtained from each patient. The authors declare that they complied with the Declaration of Helsinki code of ethics [29].

Data were entered and processed using SPSS^®^ version 20.0 for iOS (SPSS, Inc. Chicago, IL, USA). Descriptive analyses were performed. For quantitative variables, we calculated the means and standard deviations. The student’s *t*-test was performed to compare the averages between the time to healing and treatment applied. For qualitative variables, we calculated the frequency distributions and percentages. The *X*^2^ test was used to identify differences in qualitative variables. Odds ratios and the 95% confidence intervals were determined through a univariate model. The Kaplan–Meier method was used to describe the complication-free survival of the patients, and the log-rank test was used to compare this survival between the two-osteomyelitis treatment groups. Differences were considered significant at *p* < 0.05 for a confidence interval of 95%.

## 3. Results

A total of 116 patients were included in the study; 96 (82.8%) patients received surgical treatment and 20 (17.2%) medical treatment. The main baseline characteristics are summarised in Table 1.

The following surgical procedures were performed in the surgery group: 33 (34.4%) metatarsal head resections, 20 (20.8%) osteotomies, 18 (18.8%) arthroplasties, 18 (18.8%) phalangectomies, three (3.1%) exostectomies, two (2.1%) sesamoidectomies, and two (2.1%) partial calcanectomies. The antibiotics prescribed to patients who received medical treatment were as follows: co-amoxiclav, seven (35.0%); levofloxacin, six (30.0%); ciprofloxacin, three (15.0%); a combination of levofloxacin and clindamycin, two (10.0%); and vancomycin, two (10.0%).

We found no association between healing time and treatment administered for the management of osteomyelitis: the surgery group healed within 15.7 ± 9.2 weeks versus 16.4 ± 12.1 weeks in the medical group (*p* = 0.103).

A total of 101 (87.1%) patients completed all study visits. Twelve (11.3%) patients finished the study voluntarily, and three (2.6%) patients died.

**Figure 1:**
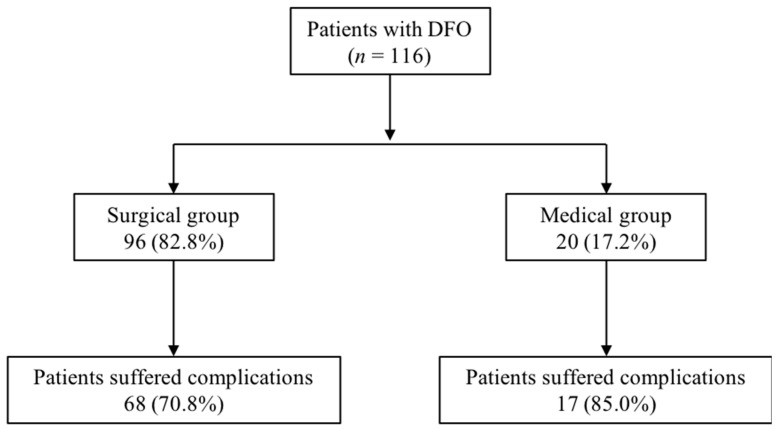
Flow chart of the distribution of complications. Abbreviation: DFO, diabetic foot osteomyelitis.

During 1 year follow-up, 85 (73.3%) patients suffered complications. Complications during follow up were similar in both groups: 68 (70.8%) patients in the surgery group showed complications versus 17 (85.0%) patients in the medical group (*p* = 0.193) (Figure 1). The most common complications in both groups were re-ulceration. The distribution of complication between both groups of treatment is shown in Table 2.

**Figure 2:**
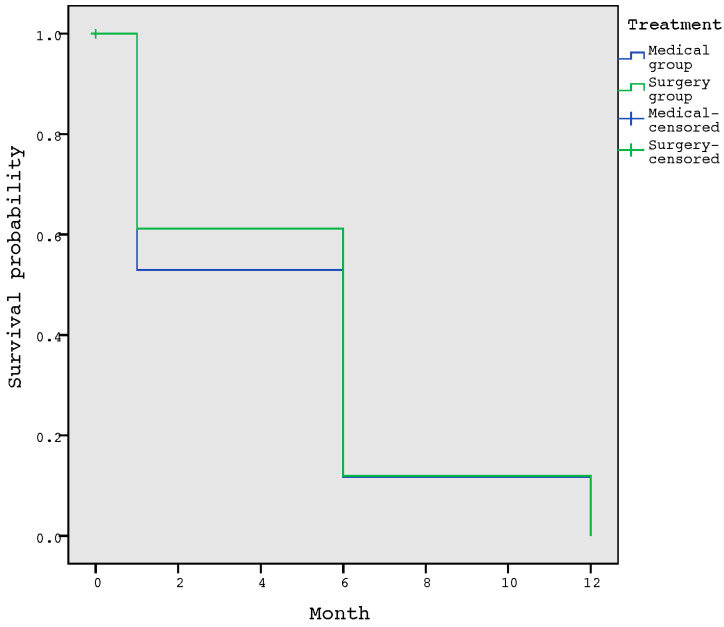
Survival function for the development of a complication according to the treatment administrated.

The probability of suffering a complication event in the first month after healing was 59.5%, and the median complication-free survival was 6 months. When comparing the survival of the complication-free time between the surgical and medical treatment of osteomyelitis, we did not observe significant differences (*p* = 0.665) (Figure 2).

## 4. Discussion

No relationship was found between healing time and the treatment administered for the management of osteomyelitis. Moreover, no differences were found between the percentages of complications that suffered from each group. Regarding the probability of suffering complications during the 1 ear follow up, we observed a clear trend in the increase in the number of complications as time progressed. In the comparison between complication-free time in patients managed with surgical versus medical treatment for osteomyelitis, we obtained similar data in terms of survival between both treatments.

Our data showing a similar duration until healing between treatment groups is consistent with a previous publication [30] that discussed that if the patient received optimal treatment (focus on effective treatment) for DFO, the response would be similar. According to our results, when a patient receives the indicated treatment for the management of DFO, the time to healing is homogeneous, showing the importance of planning adequate treatment for each case. Moreover, the first clinical trials that compared surgical versus medical treatment for the management of DFO [3], as well as our study, found no differences in median time to healing between both groups at 6 weeks (Q1 3 weeks; Q3 9 weeks) in the surgical group versus 7 weeks (Q1 5 weeks; Q3 8 weeks) in the medical group (*p* = 0.72).

The management of DFO requires an early and precise treatment after an adequate and early diagnosis, selecting appropriate antibiotic therapy and rapid determination of the patient who requires surgical treatment [5,12,31]. Regarding the selection of the best therapy to manage patients with DFO, we acknowledge that the typical profile of those patients who received medical therapy was different from those patients who received surgical treatment in our study. Unfortunately, and after decades, the selection of the best therapy for patients with DFO remains a matter of debate [32]. Nevertheless, there are published recommendations [12,15] that help clinicians select the best therapy according to the clinical presentation (e.g., soft-tissue infection or necrotising infection), vascular disease, or ulcer location and that determines if the surgery should be mandatory or not. For instance, in cases where DFO is chronic, typically, the forefoot is affected with a small ulcer, there is no PAD, there is a soft-tissue infection, and it is easy to off-load, then medical treatment is appropriate. On the contrary, in the case of a patient with acute DFO in the hindfoot, with bone exposure and soft-tissue infection, it would almost be impossible to achieve coverage of the ulcer with new tissue and resolve DFO using antibiotics only. Thus, in such cases, bone resection and soft tissue debridement are almost mandatory to obtain a resolution of infection. Furthermore, a recently published systematic review [32] that compared medical versus surgical management of DFO concluded that efficient treatment of DFO requires selection of the appropriate method according to the indication and specific characteristics of the patient.

In our study, we analysed what happened with the patients after suffering DFO in a long-term follow-up. The trend that we observed regarding the increase in the development of complications as time progressed was expected, since patients with diabetic foot, once they have suffered an ulcer and/or amputation, are classified as Risk Foot 3, according to the International Working Group of Diabetic Foot classification [33], being high-risk patients. Thus, we assumed that the longer the ulcer had healed, the more likely it was that complications would arise, since the patient spends more time exposed to the risk of developing complications.

Some previous investigations [34,35] that retrospectively compared surgical and medical treatment for the management of DFO found similar outcomes between both therapies. Game et al. [34] found similar remission rates between the surgical (78.6%) and medical group (82.3%); Lesens et al. [35] also found favourable outcomes: 80% in the surgical group versus 87% in the medical group.

A retrospective study [36] that analysed the complications associated with metatarsal head resections in patients with DFO found that re-ulceration was the most common complication after surgery. These data are the same as in our surgical group, where re-ulceration was the most common complication, followed by a new case of DFO. Although surgery could be a risk factor for re-ulceration due to the biomechanics alterations, in our study, the re-ulceration rates were similar in both groups, probably because the patients who received medical treatment continued to present peak pressures on the areas of the ulcer that did not resolve with the treatment.

However, during recent years, there is an ongoing debate about which approach to DFO management, surgical or medical, is best [3,12,13,14,15,16,17]. In our study, we compared the complication-free time of patients managed with surgical versus medical treatment during 1-year follow-up from the healing of an ulcer that was complicated with DFO. Our data on the complication-free time between both treatments are consistent with the results obtained in the first published clinical trial that compared these treatments for the management of DFO [3]. We conclude that both treatments have similar results in terms of healing rates, healing times, and short-term complications and that this similarity in the results is maintained over time, thus confirming that if the treatment to be applied in patients with DFO is properly selected, both treatments are satisfactory. Thus, the most important decision in the treatment of DFO is the adequate selection of the treatment based on the patient and ulcer characteristics.

The main limitation of our study was the difference in the sample size between both groups, with a proportion of 1:6. Moreover, we only included patients with DFO. In future research, it would be interesting to analyse a control group of patients with diabetes but without DFO and to compare their development of complications. Moreover, in future studies, it would be interesting to report other complications, such as episodes of hospitalisation, acute kidney or liver injury, or gastrointestinal side effects related to antibiotic treatment. The main strength of our study is that it is the first study to compare long-term complications after DFO. In addition, the study has an intensive follow-up programme with an important number of individuals included and a long follow-up period.

This study provides evidence to support both treatments for DFO, surgical or medical, which we showed to have similar levels of long-term success.

## 5. Conclusions

The onset of complications after healing in patients who have suffered DFO was not associated with the treatment received. Surgical and medical approaches to the management of DFO produced similar results throughout long-term follow up. During the DFO remission period in patients managed with surgery or medical treatment, we observed that a large number of patients developed a complication. Thus, patients with DFO should be considered high-risk patients even after ulcer healing. Therefore, based on our findings, we recommend follow up after ulcer healing for at least 1 year.

## Figures and Tables

**Table 1:** Baseline characteristics of the patients.

Variable	Surgery Group *n* (%)	Medical Group *n* (%)
Gender	Male: 79 (82.3) Female: 17 (17.7)	Male: 17 (85.0) Female: 3 (15.0)
Type of DM	Type 1: 11 (11.5) Type 2: 85 (88.6)	Type 1: 1 (5.0) Type 2: 19 (95.0)
PAD (mild and moderate ischemia)	39 (40.6)	9 (45.0)
Neuropathy	96 (100.0)	20 (100.0)
Location of the ulcer	Forefoot: 89 (92.7) Mindfoot: 5 (5.2) Hindfoot: 2 (2.1)	Forefoot: 18 (90.0) Mindfoot: none Hindfoot: 2 (10.0)
**Variable**	**Surgery Group** **Mean ± SD**	**Medical Group** **Mean ± SD**
Age (years)	63.1 ± 10.1	62.0 ± 10.3
DM duration (years)	17.8 ± 12.7	15.8 ± 10.0
HbA1c (%)	8.2 ± 6.5	7.8 ± 1.7
Body mass index (Kg/cm^2^)	28.3 ± 4.6	28.4 ± 8.8
Ulcer duration (weeks)	15.8 ± 34.6	15.0 ± 16.4

DM, Diabetes mellitus; PAD, peripheral arterial disease; DFO, diabetic foot osteomyelitis; SD, standard deviation; HbA1c, glycosylated haemoglobin. Bold: distinguish qualitative from quantitative variables.

**Table 2:** Surgical versus medical complications.

	Surgical Group *n* = 96				Medical Group *n* = 20			
	Visit 2	Visit 3	Visit 4	Total	Visit 2	Visit 3	Visit 4	Total
	*n* = 26	*n* = 48	*n* = 47		*n* = 8	*n* = 14	*n* = 8	
Re-ulceration	14	31	20	65	4	8	3	15
*n* (%)	(53.8)	(64.6)	(42.6)	(67.7)	(50)	(57.1)	(37.5)	(75)
New case DFO	5	9	17	31	1	1	3	5
*n* (%)	(19.2)	(18.7)	(36.2)	(32.2)	(12.5)	(7.1)	(37.5)	(25)
DFO recurrence	3	2	3	8	none	2	none	2
*n* (%)	(11.5)	(4.2)	(6.4)	(8.4)		(14.3)		(10)
Ulcer recurrence	2	3	3	8	2	1	none	3
*n* (%)	(7.7)	(6.2)	(6.4)	(8.4)	(25)	(7.1)		(15)
Other	2	2	1	5	1	none	none	1
*n* (%)	(7.7)	(4.2)	(2.1)	(5.2)	(12.5)			(5)
Soft tissue infection	none	1	none	1	none	none	none	none
*n* (%)		(2.1)		(1.1)				
Major amputation	none	none	2	2	none	none	1	1
*n* (%)			(4.3)	(2.1)			(12.5)	(5)
Death	none	none	1	1	none	2	1	3
*n* (%)			(2.1)	(1.1)		(14.3)	(12.5)	(15)

DFO, diabetic foot osteomyelitis.

## Data Availability

The data are available previous request to corresponding author.
